# Exploring the Link between Xerostomia and Oral Health in Mental Illness: Insights from Autism Spectrum Disorder, Depression, Bipolar Disorder, and Schizophrenia

**DOI:** 10.3390/healthcare12202018

**Published:** 2024-10-11

**Authors:** Rou-Jun Chen, Kuei-Hung Lai, Chun-Hung Lee, Hao-Ying Lin, Cheng-Chieh Lin, Chi-Hsiu Chen, Wei Chen, Wei-Yu Chen, Thi Thuy Tien Vo, I-Ta Lee

**Affiliations:** 1 School of Dentistry, College of Oral Medicine, Taipei Medical University, Taipei 110301, Taiwan; b202109010@tmu.edu.tw (R.-J.C.); b202109028@tmu.edu.tw (C.-H.L.); b202109011@tmu.edu.tw (H.-Y.L.); b202109083@tmu.edu.tw (C.-C.L.); b202109036@tmu.edu.tw (C.-H.C.); b202109034@tmu.edu.tw (W.C.); b202109055@tmu.edu.tw (W.-Y.C.); 2 Graduate Institute of Pharmacognosy, College of Pharmacy, Taipei Medical University, Taipei 110301, Taiwan; kueihunglai@tmu.edu.tw; 3 Traditional Herbal Medicine Research Center, Taipei Medical University Hospital, Taipei 110301, Taiwan; 4 PhD Program in Clinical Drug Development of Herbal Medicine, College of Pharmacy, Taipei Medical University, Taipei 110301, Taiwan; 5 Faculty of Dentistry, Nguyen Tat Thanh University, Ho Chi Minh City 700000, Vietnam; vtttien@ntt.edu.vn

**Keywords:** mental disorders, oral health, autism spectrum disorder, depression, bipolar disorder, schizophrenia

## Abstract

Background: The relationship between mental disorders and oral health is complex, involving behavioral, biological, and psychosocial factors. This review aims to investigate the impact of mental disorders, including autism spectrum disorder (ASD), depression, bipolar disorder, and schizophrenia, on oral health outcomes. Methods: A comprehensive review of existing literature was conducted to analyze the oral health outcomes associated with each mental disorder. The focus was on examining dietary habits, oral hygiene behaviors, physiological changes, and medication side effects that contribute to oral health issues. Results: The findings indicate that individuals with ASD often exhibit unique dietary habits and reduced oral hygiene capabilities due to sensory sensitivities, leading to a higher prevalence of dental caries and periodontal diseases. Depression and bipolar disorder are associated with physiological changes such as reduced saliva production and poor oral hygiene behaviors, increasing the risk of oral health problems. Medications used for these conditions exacerbate issues like xerostomia, further elevating the risk of dental diseases. Schizophrenia poses additional challenges, including cognitive impairments and medication side effects that hinder effective oral care, heightening susceptibility to oral diseases. Conclusions: This review highlights the specific oral health challenges associated with different mental disorders and emphasizes the need for tailored dental care strategies that integrate mental health considerations. The study contributes to the literature by demonstrating the unique oral health impacts of these disorders. However, the findings are limited by the scope of available cross-sectional data and the absence of longitudinal studies. Future research should focus on longitudinal and intervention-based studies to explore causal relationships and develop effective treatments.

## 1. Introduction

The relationship between mental disorders and oral health is increasingly recognized in healthcare research. Poor oral health not only leads to dental issues like caries and periodontal disease, but also has systemic implications. Studies show that inadequate oral hygiene can contribute to conditions such as cardiovascular disease, diabetes, and respiratory infections. In particular, periodontitis has been linked to a higher risk of atherosclerosis and cardiovascular events. Oral pathogens entering the bloodstream can also exacerbate or initiate chronic conditions in distant organs. This bidirectional relationship highlights the importance of maintaining good oral hygiene for both dental and overall health.

Understanding the impact of mental health disorders on oral hygiene is essential for evaluating broader health outcomes. Conditions such as autism spectrum disorder (ASD), depression, bipolar disorder, and schizophrenia present significant oral health challenges, directly and indirectly affecting individuals’ ability to maintain oral hygiene. While schizophrenia and bipolar disorder share etiological factors like genetic predisposition and neurotransmitter dysregulation, and depression and ASD have distinct mechanisms, this review addresses these disorders collectively to provide a comprehensive overview of their oral health impact. Co-occurring mental disorders often amplify the negative effects on oral health, as studies show that patients with comorbid conditions, such as depression and schizophrenia, are at higher risk for severe oral diseases. Factors such as cognitive impairments, medication side effects (e.g., xerostomia), and reduced self-care motivation contribute to these challenges. Research by Kisely et al. indicates that co-occurrence of mental disorders significantly increases the likelihood of dental caries and periodontal disease, highlighting the need for a multidisciplinary healthcare approach that integrates mental and dental care [1,2]. Variability in oral health behaviors across psychiatric disorders is also considered, recognizing that cognitive impairments, motivation levels, and medication side effects vary significantly between disorders like schizophrenia and mood disorders.

While the reviewed mental disorders pose risks to oral health, their specific impacts and mechanisms vary. For instance, ASD primarily affects oral health through sensory sensitivities and motor coordination difficulties, leading to challenges in maintaining routine oral hygiene. In contrast, depression is associated with reduced motivation for self-care and xerostomia due to antidepressant medications, increasing the risk of dental caries. Bipolar disorder presents a unique challenge, as manic phases may result in aggressive oral hygiene behaviors, while depressive phases lead to neglect, contributing to oral tissue damage and periodontal disease. Schizophrenia complicates oral care through cognitive impairments and medication side effects like tardive dyskinesia, affecting the execution and consistency of oral hygiene routines. These distinctions underscore the need for dental care strategies tailored to the specific challenges of each mental disorder [1,3,4]. Understanding how different psychiatric conditions uniquely impact oral hygiene practices is critical for developing integrated care strategies that address both mental and oral health simultaneously [1,5]. Integrated care involves collaborative efforts between dental and mental health professionals, including routine dental evaluations, individualized oral hygiene instructions tailored to mental health symptoms, and management of psychiatric medication side effects, such as xerostomia, through saliva substitutes or medication adjustments. 

This review contributes to the literature by synthesizing findings on the bidirectional relationship between mental health disorders and oral health, with a focus on emerging personalized and integrative treatment approaches. Digital health technologies, such as mobile apps and tele-dentistry, are becoming increasingly important, offering real-time monitoring of oral health behaviors and delivering timely feedback and reminders for dental care in individuals with mental disorders [1]. Additionally, the growing body of research on the microbiome suggests that targeting the oral microbiome could impact mental health outcomes via the gut–brain axis [6]. Pharmacological innovations are also underway, including the development of medications designed to mitigate the xerostomic side effects of psychotropic drugs, thereby reducing associated oral health risks [1,7].

Unlike previous reviews that focus on single mental health conditions, this review provides a comprehensive examination of how multiple disorders—ASD, depression, bipolar disorder, and schizophrenia—affect oral health. To advance future research, a broader scope of dental (e.g., oral health-related quality-of-life indices) and psychological measures (e.g., detailed psychiatric assessments) is needed to fully capture the intricate relationship between mental and oral health. This review underscores the distinct oral health challenges posed by each mental disorder and the systemic consequences of poor oral hygiene, advocating for integrated healthcare strategies that address these unique needs. Such a holistic approach is crucial for identifying knowledge gaps and guiding future research and clinical practices to improve oral health outcomes for individuals with mental health conditions.

Individuals with ASD often exhibit distinct behavioral patterns, such as preferences for soft or sugary foods and aversions to certain textures, which can increase the risk of dental caries and periodontal disease. Sensory sensitivities may complicate routine oral hygiene practices, like toothbrushing and flossing, for both individuals and their caregivers [8]. Additionally, motor coordination difficulties can hinder effective oral hygiene, further elevating the risk of oral health issues [9]. However, some studies indicate that with appropriate care and support, individuals with ASD may not have a higher prevalence of dental caries compared to the general population [2]. Age also plays a role, as younger individuals typically face more challenges due to behavioral and sensory issues, while older individuals can improve their oral hygiene practices with sufficient support. Both age groups benefit from dental strategies that address sensory sensitivities and motor coordination challenges [5].

Depression poses a significant challenge to oral health, often resulting in reduced motivation for self-care and increased consumption of cariogenic foods as a coping mechanism. It is closely linked to alterations in neurotransmitter levels, particularly serotonin. Commonly used medications, such as selective serotonin reuptake inhibitors (SSRIs), can lead to xerostomia, exacerbating oral health challenges. Depression may also decrease saliva production, a side effect of the condition and its treatments [10]. Xerostomia reduces saliva’s protective effects against tooth decay and oral infections, increasing the risk of these conditions in depressed individuals [11]. The cyclical nature of depression, marked by periods of heightened symptoms, complicates oral hygiene consistency, as individuals may neglect daily care routines during depressive episodes [12].

Bipolar disorder, characterized by alternating manic and depressive phases, similarly affects oral health behaviors. During manic episodes, patients may engage in excessive oral hygiene practices, potentially damaging oral tissues or wearing down enamel [3]. However, many individuals with bipolar disorder do not experience clear manic or depressive episodes, and may present with mixed features or mild mood fluctuations. This variability can disrupt regular oral hygiene behaviors, as mood instability often leads to inconsistent self-care practices [13]. Conversely, during depressive phases, neglect of oral hygiene can result in plaque buildup and a higher incidence of caries and periodontal diseases. Psychotropic medications for bipolar disorder frequently cause xerostomia, compromising oral health by decreasing saliva’s natural cleansing action and increasing vulnerability to oral pathogens [14].

In schizophrenia, cognitive impairments, negative symptoms, and the side effects of antipsychotic medications create significant challenges for maintaining oral health. Schizophrenia presents a spectrum of symptoms, including positive symptoms (hallucinations, delusions, and disorganized thinking) and negative symptoms (emotional flatness, lack of motivation, and social withdrawal). Positive symptoms typically respond better to dopamine antagonist antipsychotics like haloperidol, which primarily target dopamine D2 receptors. In contrast, negative symptoms often persist and are less responsive to traditional antipsychotics, necessitating newer atypical antipsychotics such as clozapine or olanzapine, which act on both dopamine and serotonin receptors. These differential effects influence patients’ ability to engage in self-care, including oral hygiene. Individuals with negative symptoms are more likely to experience apathy and neglect their oral care routines, despite medication [15,16]. Cognitive deficits can further impair understanding and execution of effective oral hygiene, while negative symptoms discourage regular care [17]. Antipsychotic medications are frequently associated with xerostomia, which reduces saliva flow and increases the risk of dental caries and periodontal disease. Additionally, difficulties in accessing appropriate dental care due to social stigma, financial constraints, or a lack of specialized services further exacerbate these issues for individuals with schizophrenia [18]. 

Despite these known associations, the current literature has substantial limitations. Many studies are cross-sectional, which restricts the ability to infer causality between mental disorders and poor oral health outcomes [3,9]. Additionally, there is significant variability in study populations, diagnostic criteria, and measures of oral health outcomes, complicating the generalization of findings across different settings [19]. Furthermore, much existing research fails to account for potential confounding factors, such as socioeconomic status and access to dental care, both of which may influence oral health outcomes in individuals with mental disorders [8]. Socioeconomic status significantly impacts access to dental care and oral health. Studies indicate that individuals from lower socioeconomic backgrounds are more likely to experience poorer oral health outcomes, including higher rates of dental caries and periodontal disease, due to limited access to preventive care and education. For example, Guarnizo-Herreño et al. found that individuals from lower socioeconomic status groups were 1.7 times more likely to suffer from untreated dental decay compared to those from higher socioeconomic groups [20]. Similarly, Locker reported that socioeconomic deprivation is strongly associated with worse oral health, particularly in communities with fewer dental health resources [21]. This disparity is exacerbated in populations with mental disorders, where lower socioeconomic status may limit access to specialized mental and dental healthcare services, further worsening oral health outcomes. To better understand these complex interactions, longitudinal studies are needed to establish causal relationships more definitively [2]. In this review, we account for psychological variables such as medication use, symptom severity, and cognitive impairments to reduce potential confounding effects on oral health outcomes. By controlling for these factors, we provide a more accurate representation of how mental health disorders affect oral hygiene behaviors.

Integrating mental and oral health care is essential for improving overall health outcomes in individuals with mental disorders. By addressing the unique needs of these populations through targeted interventions and comprehensive care strategies, healthcare providers can enhance quality of life and reduce the burden of oral disease. Although this review is not systematic, we aim to provide a comprehensive overview of the current literature on the relationship between mental health disorders and oral health. We include a range of peer-reviewed studies that focus on how ASD, depression, bipolar disorder, and schizophrenia impact oral health. The sample size for this review was based on studies that specifically examined the intersection of oral and mental health outcomes, adhering to clear diagnostic criteria for both to ensure reliability and relevance. The selected studies contributed to understanding the broader implications of these mental disorders on oral hygiene behaviors and outcomes. Our goal was to synthesize findings from diverse sources to highlight key trends, gaps, and areas for future research. By doing so, we aim to reflect the current state of knowledge while addressing limitations in the existing literature. 

## 2. ASD and Oral Health

### 2.1. Dental Caries in Individuals with ASD

The prevalence of dental caries in individuals with ASD has been a significant focus of recent oral health research, with varying results contributing to an ongoing debate about whether these individuals are at higher risk compared to the general population. Research supporting a higher prevalence often cites factors such as unique dietary habits, sensory sensitivities, and difficulties with oral hygiene practices. For instance, individuals with ASD may prefer soft, sugary foods due to sensory sensitivities, increasing their risk of dental caries [22]. A study by Ferrazzano et al. found that children with ASD had higher rates of dental caries, attributed to both their dietary preferences and challenges in effective oral hygiene due to behavioral issues [22]. Other studies, like those conducted by Marshall et al., corroborate these findings, highlighting the fact that heightened sensory aversions make routine dental care, including brushing and flossing, particularly difficult [5].

Several studies, including those by Jaber [23] and Babu and Roy [24], indicate that individuals with autism are more prone to dental caries than their neurotypical peers. This increased risk is primarily due to sensory sensitivities, preferences for sweet and soft foods, and challenges in maintaining oral hygiene due to motor skill difficulties. Both studies note that individuals with autism may hold food in their mouths or consume beverages other than water, further elevating their risk of dental caries. Delays in motor development and coordination issues also impair their oral hygiene practices.

Conversely, some studies challenge the idea that individuals with ASD have a higher prevalence of dental caries. Morales-Chávez et al. [25] reported no significant difference in caries rates between children with ASD and those without, possibly due to vigilant oral care by caregivers or dietary restrictions limiting sugary food intake. Similarly, Delli et al. found that individuals with ASD may consume fewer sugary snacks and gluten-containing foods, leading to fewer cavities [26]. Recent data from studies like Loo et al. suggest that children with ASD who receive specialized dental care tailored to their needs may not show an increased prevalence of caries, underscoring the importance of personalized dental care strategies [27]. This variation in findings may also be influenced by the level of support and education provided to caregivers, the availability of specialized dental services, and regional differences in dietary habits and healthcare systems.

### 2.2. Periodontal Disease and ASD

Periodontal disease is a significant concern for individuals with ASD, primarily due to challenges in maintaining proper oral hygiene. A study by Tsai et al. indicated that adolescents with ASD are more prone to periodontal diseases than their neurotypical peers, mainly due to inadequate oral hygiene practices [28]. These challenges often stem from the sensory and behavioral characteristics of ASD, which may include resistance to toothbrushing and flossing, due to sensory sensitivities. Additionally, a lack of manual dexterity can lead to improper brushing techniques, increasing the risk of plaque accumulation and periodontal disease [27]. Loo et al. found that children with ASD experience more significant periodontal issues due to these compounding factors, but these issues could be mitigated with early and consistent dental interventions and caregiver education [27].

Many studies consistently show that individuals with autism have a higher probability of developing periodontitis and related diseases due to poor oral hygiene environments [28]. However, no research has demonstrated a genetic link between autism and an increased prevalence of periodontal disease, suggesting that the higher prevalence is likely due to behavioral factors.

Medications used to manage ASD-related symptoms, such as antipsychotics and anticonvulsants, can contribute to xerostomia, increasing the risk of periodontal disease by creating an environment conducive to bacterial growth. To improve oral health outcomes and reduce the prevalence of periodontal disease in individuals with ASD, comprehensive care plans are essential. These plans should address not only medication side effects like xerostomia, but also sensory sensitivities and behavioral challenges unique to this population [29]. Stein et al. [30] emphasize the importance of such integrated approaches in managing both oral health and ASD-related challenges. 

### 2.3. Other Oral Health Issues in ASD (Open Bite, Xerostomia)

In addition to dental caries and periodontal disease, individuals with ASD are at risk for other oral health issues, such as open bite and xerostomia. Open bite is commonly observed in individuals with ASD and can result from behaviors like tongue thrusting, prolonged pacifier use, or other parafunctional habits often employed as self-soothing techniques [31]. A study by Watanabe et al. found a significant association between open bite and ASD, attributing many cases to a combination of behavioral factors and medication side effects, such as muscle stiffness from psychotropic drugs [29]. The presence of an open bite affects aesthetics and has implications for speech and eating, highlighting the need for early orthodontic evaluation and intervention. This aligns with findings from Watanabe’s article, which concluded that individuals with autism often experience open bite due to the interaction between psychiatric medications and dental treatment [29].

Xerostomia, or dry mouth, is a common issue among individuals with ASD, often resulting from the side effects of various medications, including antipsychotics. While xerostomia is significant, it is not the primary concern for all individuals with mental health disorders. Behavioral challenges, reduced motivation for self-care, and cognitive impairments associated with these conditions often have a greater impact on oral health. Reduced saliva production diminishes the mouth’s natural defenses, increasing the risk of dental caries and periodontal disease. The lack of saliva impairs mechanical cleansing in the oral cavity and reduces the buffering capacity against acids produced by bacteria, creating an environment conducive to dental caries and periodontal pathogens [32]. Adults with ASD are particularly vulnerable to xerostomia due to the cumulative effects of long-term medication use, with one study finding a prevalence of 32% among individuals with autism, indicating a higher incidence compared to the general population. Regular dental evaluations and targeted oral hygiene practices are necessary to mitigate these risks [33]. Additionally, another study reported a prevalence of 21.7%, further underscoring this increased risk [34].

To address these oral health challenges, tailored dental care strategies that consider the unique behavioral and sensory needs of individuals with ASD are essential. Interventions such as desensitization techniques, caregiver education, and specialized dental care routines can significantly improve oral health outcomes. Collaborative efforts among dental professionals, caregivers, and behavioral specialists are critical for developing comprehensive care plans that effectively address both the oral health needs and behavioral characteristics associated with ASD.

## 3. Depression and Oral Health

### 3.1. Impact of Depression on Oral Hygiene and Health

Depression significantly impacts oral health by affecting both oral hygiene behaviors and physiological conditions that lead to adverse outcomes. Individuals with depression often experience reduced motivation and energy levels, which can severely impact their ability to maintain regular oral hygiene routines, such as brushing and flossing. This neglect of oral care leads to plaque accumulation and an increased risk of dental caries and periodontal diseases. A systematic review by Kisely et al. demonstrated that individuals with depressive disorders have significantly higher rates of dental caries and periodontal disease compared to those without mental health disorders. The study attributed these findings to both decreased oral hygiene practices and physiological changes associated with depression, such as altered immune responses that impair the body’s ability to fight oral infections [1].

Depression is also linked to physiological conditions that further compromise oral health. For instance, antidepressant medications, such as SSRIs and tricyclic antidepressants, are known to cause xerostomia, or dry mouth, which significantly reduces saliva production. Saliva is crucial for maintaining oral health because it neutralizes acids produced by bacteria, provides antimicrobial proteins, and aids in the remineralization of tooth enamel. A reduction in saliva flow increases the risk of dental caries and other oral infections. According to Wolff et al., patients on antidepressants, particularly tricyclic antidepressants and SSRIs, experience significantly reduced salivary flow rates, which leads to a higher prevalence of dental caries and periodontal diseases [7]. Sreebny and Schwartz further support these findings, noting that chronic dry mouth in patients on long-term antidepressant medications contributes to increased dental caries and other oral health issues, due to the diminished protective effects of saliva [35].

Moreover, depression can alter pain perception, increasing sensitivity to oral discomfort, which may discourage individuals from seeking routine dental care or maintaining consistent oral hygiene practices. Kisely et al. found that individuals with depressive symptoms were more likely to report higher levels of dental anxiety and pain sensitivity, correlating with poorer oral health outcomes due to avoidance of dental visits and inconsistent care routines [8]. This avoidance creates a cycle of worsening oral conditions and increased reluctance to seek care, leading to the progression of untreated oral health issues.

Additionally, behaviors associated with depression, such as teeth grinding (bruxism) or jaw clenching, can exacerbate dental problems and lead to temporomandibular joint disorders (TMJDs). This increased muscle tension and stress contribute to pain and functional impairment in the temporomandibular joint and related structures, further complicating oral health [36,37].

### 3.2. Connection between Depression, Nutrition, and Oral Health

Depression not only affects oral hygiene behaviors, but also influences dietary habits, which can directly impact oral health. Individuals with depression may engage in unhealthy eating patterns, often neglecting general nutrition. This can result in deficiencies in essential vitamins and minerals that are important for maintaining oral health. For example, a lack of vitamins C and D, calcium, and phosphorus can compromise the integrity of the teeth and supporting structures, making them more susceptible to decay and periodontal disease [38]. A study by Jacka et al. demonstrated that individuals with depression often have lower intakes of essential nutrients, which correlates with increased rates of periodontal disease and tooth loss, due to compromised immune function and poor oral health [39]. This issue is not unique to depression. Nutritional deficiencies are commonly observed across a range of mental health conditions, including ASD, bipolar disorder, and schizophrenia. Individuals with these disorders also frequently exhibit poor dietary habits, which lead to a lack of essential vitamins and minerals, further impacting their oral health. The findings from Jacka et al. can be applied to these populations as well, as inadequate nutrition compromises immune function and oral health integrity, increasing the risk of periodontal disease and tooth loss across these conditions [39].

Depression-induced nutritional deficiencies can also impair immune function, reducing the body’s ability to respond effectively to oral infections. A weakened immune system allows for the proliferation of pathogenic bacteria in the oral cavity, contributing to the development and progression of periodontal disease. A study by Okoro et al. explored the relationship between depression and periodontal disease, emphasizing that poor nutritional status, common in individuals with depression, exacerbates periodontal conditions and complicates their management [40]. For instance, the inflammatory response in the gums can become more severe, leading to faster progression of periodontal disease and increased tooth loss.

In summary, depression affects oral health through behavioral neglect, poor nutrition, and compromised immune function. Addressing these issues requires a comprehensive approach that includes mental health management, dietary counseling, and enhanced dental care strategies. A holistic approach, combining dental and psychological care, is essential to mitigate the adverse effects of depression on oral health.

## 4. Bipolar Disorder and Oral Health

### 4.1. Oral Health Complications in Bipolar Disorder

Individuals with bipolar disorder face unique oral health challenges stemming from the distinct phases of their condition. During manic episodes, increased energy, impulsivity, and restlessness can lead to behaviors such as excessive toothbrushing, causing enamel abrasion and gum damage. Manic phases are also associated with high-risk behaviors, including substance abuse and irregular eating patterns, which can result in nutritional deficiencies that weaken oral tissues and increase susceptibility to infections and diseases.

Conversely, during depressive episodes, individuals with bipolar disorder may experience a significant lack of motivation for self-care, leading to poor oral hygiene practices, such as infrequent tooth brushing and flossing. This neglect leads to plaque accumulation, increasing the risk of dental caries and periodontal disease progression. A study by Kisely et al. demonstrated that individuals with severe mental illnesses, including bipolar disorder, have a higher prevalence of dental diseases, largely due to inconsistent oral hygiene practices influenced by their fluctuating mental state [1]. The study highlights the fact that during depressive phases, individuals may avoid dental care entirely, exacerbating oral health problems and creating a cycle of neglect and worsening conditions.

Clinically, the erratic behavior associated with bipolar disorder, particularly during manic episodes, can lead to either overzealous oral care or complete neglect. For example, a study found that during manic episodes, some patients engage in repetitive or excessive brushing and flossing, causing damage to the oral tissues. In contrast, depressive phases are characterized by a decline in energy and interest, leading to significant lapses in oral hygiene, allowing dental plaque to build up, increasing the risk for both dental caries and periodontal diseases [41].

### 4.2. Medication Side Effects and Oral Health

Medications used to manage bipolar disorder, such as mood stabilizers (e.g., lithium) and antipsychotics, often have side effects that can adversely impact oral health. Lithium, a common mood stabilizer, is associated with xerostomia, a condition that significantly reduces saliva production. Saliva is crucial for maintaining oral health, as it neutralizes acids produced by bacteria, provides antimicrobial properties, and facilitates the remineralization of tooth enamel. Reduced saliva production increases the risk of dental caries and oral infections, due to a compromised oral environment. A study by Friedlander et al. found that patients on lithium therapy frequently experience xerostomia, which diminishes the protective effects of saliva and increases their vulnerability to oral health problems [42].

Antipsychotic medications prescribed to manage the symptoms of bipolar disorder can also cause xerostomia and, in some cases, sialorrhea (excessive drooling), depending on the specific medication and dosage. Xerostomia exacerbates the risk of caries and periodontal diseases by reducing the natural cleansing action of saliva and its buffering capacity against acids. On the other hand, sialorrhea can lead to irritation of the skin around the mouth, discomfort, and social embarrassment, indirectly affecting oral hygiene practices. These side effects necessitate dental professionals remaining vigilant and collaborating closely with patients and their healthcare providers to implement preventive dental care strategies and educate patients on managing these symptoms [42].

Furthermore, anticonvulsant medications such as valproate, which are often prescribed to manage mood swings in bipolar disorder, can lead to gingival hyperplasia, a condition characterized by an overgrowth of gum tissue [42,43,44]. This overgrowth makes oral hygiene practices more challenging and increases the risk of periodontal disease. Gingival hyperplasia can create pockets where plaque and bacteria accumulate, leading to inflammation and infection of the gums. Research by Scully and Bagan suggests that patients taking these medications should have regular dental check-ups to monitor and mitigate these adverse effects effectively [44]. They emphasize the importance of maintaining rigorous oral hygiene and possibly adjusting medication or dosages in consultation with healthcare providers to reduce the risk of severe oral health issues.

Clinical data further suggest a direct correlation between the type of medication used in bipolar disorder management and the prevalence of specific oral health conditions. For example, studies indicate that patients on long-term lithium therapy have a higher incidence of xerostomia, leading to dental caries and periodontal disease. Meanwhile, those on anticonvulsants like valproate show increased rates of gingival overgrowth [42,44]. This underscores the necessity for dental professionals to be aware of these medication-related side effects and work proactively with patients to develop comprehensive oral health management plans tailored to address these specific challenges.

In summary, managing oral health in individuals with bipolar disorder requires a multifaceted approach that includes awareness of behavioral patterns associated with different mood phases and careful management of medication side effects. Regular dental visits, patient education, and interdisciplinary collaboration between dental and mental health professionals are essential to mitigate the adverse effects of bipolar disorder on oral health.

## 5. Schizophrenia and Oral Health

### 5.1. Oral Hygiene Challenges in Schizophrenia

Individuals with schizophrenia often face significant challenges in maintaining oral hygiene due to cognitive impairments and medication side effects. Cognitive impairments, such as difficulties with memory, attention, and executive functioning, can hinder the ability to consistently perform basic oral care tasks like brushing and flossing. This can lead to plaque buildup, tooth decay, and gum disease. A study by Persson et al. demonstrated that patients with schizophrenia are at an increased risk of oral diseases due to a combination of cognitive challenges and medication-induced dry mouth, underscoring the need for tailored dental interventions and regular dental check-ups [45].

Antipsychotic medications, such as clozapine and olanzapine, are commonly prescribed for schizophrenia and frequently cause xerostomia, which reduces saliva production. Saliva is critical for neutralizing acids and protecting against tooth decay, so its reduction increases the risk of dental caries and periodontal disease. Another study confirmed that antipsychotic medications, including haloperidol and risperidone, are associated with tardive dyskinesia—a condition characterized by involuntary, repetitive movements. Tardive dyskinesia can significantly impair a patient’s ability to perform routine oral hygiene tasks, further increasing the risk of dental caries and periodontal disease. Tarsy and Baldessarini’s study highlighted the fact that tardive dyskinesia complicates oral care due, to these involuntary movements, emphasizing the need for dental professionals to consider these challenges when treating patients with schizophrenia [46].

In addition to these challenges, a study by Denis et al. found that individuals with schizophrenia have an oral health profile significantly affected by both cognitive impairments and the side effects of medications [47]. This profile includes difficulties in performing adequate oral hygiene due to a lack of coordination and the impact of medications that affect saliva production, such as antipsychotics and antidepressants. This further highlights the need for specialized dental care strategies tailored to the unique needs of these patients.

### 5.2. Prevalence of Periodontal Disease and Dental Caries

The prevalence of periodontal disease and dental caries is significantly higher among individuals with schizophrenia compared to the general population. This increased prevalence is attributed to multiple factors, including poor oral hygiene, medication side effects, and barriers to accessing dental care. Cognitive impairments, such as reduced attention and memory, often hinder the ability to maintain regular oral hygiene routines.

Medications commonly prescribed to manage schizophrenia, particularly antipsychotics, frequently lead to xerostomia, which reduces saliva flow and increases the risk of oral health issues such as dental caries and periodontal disease. Studies by Stiefel et al. and Angelillo et al. highlight a higher prevalence of untreated dental caries and severe periodontal disease in individuals with schizophrenia, emphasizing the need for specialized dental care and regular check-ups tailored to the challenges faced by these patients [4,48].

Additionally, Denis et al. point out that side effects, such as xerostomia and tardive dyskinesia, further complicate oral hygiene maintenance, reinforcing the importance of integrating mental and oral healthcare for individuals with schizophrenia [47].

### 5.3. Psychosocial Factors Affecting Oral Health in Schizophrenia

Psychosocial factors such as social stigma, economic barriers, and limited access to appropriate dental care significantly impact the oral health of individuals with schizophrenia. The stigma associated with mental illness often leads to discrimination and social isolation, reducing the likelihood that these individuals will seek or receive adequate dental care. Financial constraints also limit access to dental services, especially for those without insurance coverage that includes dental care.

Furthermore, there is often a lack of dental professionals trained to address the specific needs of psychiatric patients, which complicates access to care. A study by Kisely et al. found that individuals with severe mental illnesses, including schizophrenia, often face numerous barriers to accessing dental care, such as social stigma, financial difficulties, lack of transportation, and insufficient social support [1]. These barriers contribute to poorer oral health outcomes among this population. The study emphasizes the necessity for integrated healthcare approaches that address both mental and oral health needs to provide comprehensive care for individuals with schizophrenia.

Denis et al. also discuss how psychosocial factors, including stigma and limited access to dental care, exacerbate existing dental problems [47]. They note that many people with schizophrenia may not prioritize oral health due to a lack of awareness and understanding of its importance, compounded by their mental health challenges. This finding underscores the need for healthcare providers to develop strategies that reduce barriers and encourage regular dental visits and proper oral hygiene practices.

In conclusion, individuals with schizophrenia face a range of challenges that can severely impact their oral health. Addressing these issues requires a comprehensive approach that considers cognitive, medication-induced, and psychosocial factors, ensuring these patients receive the necessary support to maintain good oral health.

## 6. Discussion

The findings of this review emphasize the profound impact that mental illnesses such as ASD, depression, bipolar disorder, and schizophrenia can have on oral health. Each condition presents unique challenges that affect not only the ability of individuals to maintain proper oral hygiene, but also their overall susceptibility to dental diseases and their access to dental care. For instance, individuals with bipolar disorder may experience inconsistent oral care due to the cyclic nature of manic and depressive episodes, where manic phases may lead to overzealous brushing and depressive phases to oral neglect. Schizophrenia introduces additional barriers, such as cognitive impairments and medication-induced tardive dyskinesia, which complicate routine oral hygiene practices. The relationship between mental health and oral health is complex and multifaceted, involving behavioral, cognitive, and physiological factors, as well as social and economic barriers. In bipolar disorder, the alternation between manic over-care and depressive neglect further complicates oral health outcomes. Meanwhile, in schizophrenia, involuntary movements caused by tardive dyskinesia from long-term antipsychotic use present a unique challenge that is distinct from other mental illnesses. To further illustrate these challenges, Table 1 presents a summary of key studies, highlighting the oral health issues associated with different mental health disorders, along with the study findings. By exploring these key studies, we can better understand the critical need for tailored dental care strategies and integrated healthcare approaches that address the full spectrum of challenges faced by these populations (Table 2).

This table summarizes the key oral health issues associated with different mental health disorders, and presents the main findings from relevant studies. The table highlights how conditions such as ASD, depression, bipolar disorder, and schizophrenia impact oral health through behavioral patterns, medication use, and access to healthcare services, while also noting the variability in outcomes across studies. This comparison provides insights into the need for personalized dental care strategies and further research on mental health and oral health interactions.

This table provides a summary of oral health issues, potential causes, and suggested interventions across various mental disorders, including ASD, depression, bipolar disorder, schizophrenia, and anxiety/depressive disorders. Each mental disorder presents unique oral health challenges, such as dental caries, periodontal disease, xerostomia, and others. The suggested interventions are specifically tailored to address the unique behavioral, cognitive, and physiological factors associated with each condition. The references included support the relationship between psychiatric disorders and oral health outcomes, emphasizing the importance of integrated care strategies. This table highlights the need for distinct approaches in dental care for individuals with mental health disorders, ensuring that their specific needs are addressed through specialized and interdisciplinary interventions.

Individuals with ASD often face significant oral health challenges, primarily due to behavioral factors, sensory sensitivities, and unique dietary preferences. Research indicates that individuals with ASD are more likely to experience dental caries and periodontal diseases compared to their neurotypical peers. This increased risk is largely attributed to difficulties in maintaining regular oral hygiene practices, as well as a preference for foods that are high in sugar and low in nutritional value, which contribute to a higher incidence of dental problems [22,24]. The sensory sensitivities associated with ASD can make routine oral hygiene practices, such as tooth brushing and flossing, uncomfortable or distressing, leading to a lack of compliance and increased plaque accumulation [5,23]. Additionally, motor coordination difficulties, which are common in individuals with ASD, further hinder effective oral hygiene, exacerbating the risk of oral health issues. 

However, the literature also presents a more nuanced view. Some studies suggest that individuals with ASD may not necessarily have a higher prevalence of dental caries if they receive proper care and tailored dental interventions. These studies argue that with adequate support from caregivers and access to specialized dental services, individuals with ASD can achieve oral health outcomes comparable to those of the general population [25,26]. This variability in findings may be influenced by factors such as the level of caregiver support, regional differences in healthcare systems, and the availability of specialized dental services. Therefore, it is essential to consider the individual needs of each patient and to develop personalized dental care strategies that address the unique behavioral and sensory challenges faced by individuals with ASD. 

The literature presents conflicting findings regarding the oral health of individuals with ASD. While some studies suggest a higher prevalence of dental caries and periodontal diseases due to behavioral and sensory challenges [22,23], other research argues that individuals with ASD may not exhibit significantly worse oral health outcomes if proper dental care and support are provided [24,26]. For instance, some studies that show no significant difference in caries rates attribute these outcomes to enhanced caregiver support, access to specialized dental services, or restrictive diets that limit cariogenic foods [25,27]. These inconsistencies likely stem from variations in study design, sample size, geographic differences in healthcare access, and the heterogeneity of ASD itself. Moreover, socioeconomic factors play a critical role in influencing the oral health outcomes of individuals with ASD and other mental health conditions. Access to specialized dental care, the ability to afford routine check-ups, and the availability of caregiver support are often closely tied to a family’s socioeconomic status [40]. Studies have shown that individuals from lower socioeconomic backgrounds may have limited access to preventive dental care and face additional barriers in obtaining specialized services, which can exacerbate oral health disparities in vulnerable populations [49,50]. The intersection of mental health challenges and socioeconomic constraints can further complicate care, as individuals with limited financial resources may prioritize other health needs over dental care [21]. This highlights the need for healthcare systems to integrate accessible, affordable dental services that are sensitive to the unique needs of these populations, ensuring that socioeconomic disparities do not worsen oral health outcomes [20]. Meta-analyses and larger studies provide strong evidence for the link between psychiatric disorders and poor oral health outcomes. These studies emphasize the need for longitudinal and interventional research to better understand the causal relationships and to develop effective, integrated care strategies that improve both mental and oral health. Larger studies and meta-analyses have confirmed the significant link between psychiatric disorders and oral health. For instance, a systematic review by Kisely et al. [1] demonstrated that individuals with severe mental illness, including schizophrenia and bipolar disorder, have an increased prevalence of periodontal disease and dental caries compared to the general population. Similarly, Grinshpoon et al. [51] identified substantial oral health disparities among inpatients with schizophrenia, primarily due to the side effects of antipsychotic medications, which exacerbate conditions such as xerostomia and tardive dyskinesia. These findings underscore the need for integrated mental and dental healthcare approaches to mitigate these oral health disparities. However, a closer look at key studies reveals conflicting results, especially in populations with ASD, where some studies report a higher prevalence of oral health issues, while others suggest comparable outcomes to the general population when proper dental care is provided [22,26,33,34]. These discrepancies suggest that future research should focus on larger, longitudinal studies to account for regional variations and confounding factors. Many of the studies reviewed in this manuscript may have overlooked these critical socioeconomic factors, leading to inconsistent findings across different populations. For example, the availability of resources such as specialized dental care and caregiver support is highly variable, and without accounting for these factors, the true impact of ASD on oral health may be under- or overestimated. For example, studies conducted in regions with well-established dental care systems and strong caregiver support report better oral health outcomes, whereas studies from regions with limited resources highlight more severe oral health issues in this population [27,28]. Many of the studies reviewed in this manuscript may have overlooked critical socioeconomic factors, such as access to specialized dental care and caregiver support, leading to inconsistent findings across different populations. It is crucial to account for these variables to accurately assess the true impact of psychiatric disorders on oral health outcomes. The conflicting findings suggest that generalizations about the oral health of individuals with ASD should be made with caution [22,24]. Clinically, these findings underscore the need for personalized care strategies that address the unique behavioral, sensory, and socio-economic backgrounds of each patient. A one-size-fits-all approach is inadequate to address the complex needs of individuals with ASD. Furthermore, the variability in outcomes highlights the necessity of more longitudinal and controlled studies to better understand how different interventions, healthcare settings, and individual patient characteristics influence oral health outcomes [33,34]. These studies would provide clearer guidance for the development of effective, individualized dental care strategies [26,34].

Depression significantly impacts oral health, through its effects on self-care behaviors and physiological changes that increase susceptibility to dental diseases. Individuals with depression often experience a reduction in motivation and energy levels, which can severely affect their ability to maintain regular oral hygiene routines, such as brushing and flossing. This neglect of oral care leads to the accumulation of plaque and an increased risk of dental caries and periodontal diseases [8,38]. Furthermore, depression is frequently associated with xerostomia, or dry mouth, a condition that is often exacerbated by antidepressant medications such as SSRIs and tricyclic antidepressants. Xerostomia reduces saliva production, which is crucial for maintaining oral health by neutralizing acids produced by bacteria, providing antimicrobial proteins, and aiding in the remineralization of tooth enamel. Reduced saliva flow can significantly increase the risk of dental caries and other oral infections [7,35].

Depression can also alter pain perception, increasing sensitivity to oral discomfort, which may discourage individuals from seeking routine dental care or maintaining consistent oral hygiene practices. Studies have shown that individuals with depressive symptoms are more likely to report higher levels of dental anxiety and pain sensitivity, which correlates with poorer oral health outcomes due to avoidance of dental visits and inconsistent oral care practices [8]. This avoidance further compounds the negative impact of depression on oral health, creating a cycle of worsening oral conditions and increased reluctance to seek care. In addition, the cyclical nature of depression, characterized by periods of heightened symptoms, can lead to intermittent neglect of oral hygiene, further complicating oral health management for individuals with depression.

Bipolar disorder presents its own set of challenges to oral health, largely due to the alternating phases of mania and depression that characterize the condition. During manic episodes, individuals may exhibit behaviors such as excessive or aggressive tooth brushing, which can lead to enamel abrasion, gum damage, and other oral health issues [41]. Additionally, manic phases are often associated with high-risk behaviors, including substance abuse and irregular eating patterns, which can further contribute to poor oral health outcomes. For example, neglecting regular meals can lead to nutritional deficiencies, weakening the oral tissues and increasing susceptibility to infections and diseases. Conversely, during depressive episodes, individuals with bipolar disorder may experience a significant lack of motivation for self-care, leading to poor oral hygiene practices, such as infrequent tooth brushing and flossing. This neglect results in plaque accumulation, which increases the risk of dental caries and the progression of periodontal disease [8]. Moreover, the medications used to manage bipolar disorder, including mood stabilizers like lithium and antipsychotics, often have side effects that impact oral health. Lithium, for instance, is commonly linked to xerostomia, which increases the risk of dental caries and oral infections by reducing saliva production [42]. In contrast, some antipsychotic medications may lead to sialorrhea (excessive drooling), which can further complicate oral hygiene routines and exacerbate the risk of oral health problems, particularly during manic or depressive episodes when patients may be less attentive to dental care [44].

Individuals with schizophrenia face significant oral health challenges due to a combination of cognitive impairments, negative symptoms, and the side effects of antipsychotic medications. Cognitive deficits, such as difficulties with memory, attention, and executive functioning, can impair an individual’s ability to perform effective oral hygiene routines, leading to plaque buildup, tooth decay, and gum disease [45,47]. Negative symptoms, such as lack of motivation or interest, further discourage regular care, resulting in poor oral health outcomes. In schizophrenia, xerostomia resulting from antipsychotics, compounded by tardive dyskinesia, further impairs oral hygiene routines, increasing risks of dental diseases [12,51]. Tardive dyskinesia, a side effect of prolonged use of antipsychotic medications characterized by involuntary, repetitive movements, can also impair a patient’s ability to perform routine oral hygiene tasks, further increasing the risk of dental diseases [46]. Moreover, individuals with schizophrenia often encounter barriers to accessing dental care, such as social stigma, economic constraints, and a lack of dental professionals trained to address the specific needs of psychiatric patients. These barriers contribute to poorer oral health outcomes among this population [1]. The social stigma associated with mental illness often leads to discrimination and social isolation, reducing the likelihood that these individuals will seek or receive adequate dental care. Financial constraints also limit access to dental services, especially for those without insurance coverage that includes dental care. Furthermore, there is often a lack of dental professionals trained to address the specific needs of psychiatric patients, which complicates access to care [47].

Despite the significant challenges faced by individuals with mental illnesses in maintaining oral health, the literature also highlights opportunities for improving care. Tailored dental care strategies that consider the unique behavioral, cognitive, and physiological challenges faced by these populations are essential for improving oral health outcomes. To provide a clearer understanding of these strategies, a set of specific recommendations and guidelines for healthcare providers and patients is summarized in Table 3. Evidence-based strategies for improving oral health in individuals with mental health disorders include a combination of behavioral, pharmacological, and systemic interventions. For individuals with ASD, desensitization techniques and sensory-friendly dental environments have proven effective in reducing anxiety during dental visits [5,30]. In addition to regular dental check-ups for individuals with ASD, it is essential to ensure that dental providers are educated on how to work with neurodivergent populations. Providers who are knowledgeable about the sensory sensitivities, behavioral challenges, and communication needs of ASD patients can create a more supportive and effective care environment. Education programs and specialized training for dental providers can equip them with strategies to manage sensory sensitivities, provide behavior guidance, and effectively communicate with both patients and caregivers. Without such training, the goals of regular dental check-ups may not be met, as neurodivergent individuals might not receive appropriate care that aligns with their specific needs. 

This table provides specific recommendations and guidelines for healthcare providers and patients to address oral health challenges in individuals with mental disorders. It highlights tailored interventions for common issues such as xerostomia, dental caries, and periodontal disease, emphasizing the importance of interdisciplinary collaboration, specialized care environments, and daily oral hygiene practices.

Tailored dental education programs focusing on neurodiversity should be an integral part of intervention strategies for improving oral health in this population. Behavioral therapy and caregiver education can also help improve daily oral hygiene routines, especially for those with sensory sensitivities and motor coordination difficulties [26]. For individuals with depression and bipolar disorder, integrated healthcare approaches that combine mental health support with regular dental check-ups are critical [9]. The use of saliva substitutes and fluoride treatments can mitigate the effects of xerostomia, a common side effect of psychiatric medications [35]. Encouraging patients to maintain regular dental appointments during depressive phases and implementing motivational strategies can enhance oral hygiene adherence [11].

In patients with schizophrenia, cognitive impairments and medication side effects, such as xerostomia and tardive dyskinesia, complicate oral health care [45]. Therefore, frequent professional dental cleanings and saliva stimulants are essential [48]. Collaboration between mental health professionals and dentists is key to addressing both cognitive challenges and physical barriers to oral care [1]. The use of specialized dental tools and techniques, such as electric toothbrushes with larger handles or modified flossing devices, may also improve oral hygiene outcomes [41]. By applying these targeted interventions, healthcare providers can significantly enhance the oral health and overall well-being of individuals with mental health conditions.

For individuals with ASD, interventions such as desensitization techniques, caregiver education, and specialized dental care routines can significantly improve oral health outcomes. For those with depression or bipolar disorder, integrated care approaches that include mental health management, dietary counseling, and enhanced dental care strategies are crucial for mitigating the adverse effects of these conditions on oral health. For individuals with schizophrenia, reducing barriers to care, increasing awareness of the importance of oral health, and training dental professionals to manage the unique needs of psychiatric patients are critical steps toward improving oral health outcomes.

This review highlights the critical need for integrated healthcare approaches that address both mental and oral health. Although many studies support the link between mental health disorders and poor oral health outcomes, discrepancies in the prevalence of conditions like dental caries and periodontal disease persist across populations. These inconsistencies often stem from variations in study designs, sample sizes, and confounding factors such as access to dental care and socioeconomic status [22,23,25,27,40]. Additionally, the reliance on cross-sectional data limits the ability to infer causality between mental disorders and oral health outcomes. Longitudinal studies underscore the importance of understanding the progression of oral health challenges in individuals with mental health disorders. These studies provide critical insights into how long-term psychiatric treatments affect oral health, highlighting the necessity of preventive care and early interventions to mitigate these effects over time. Variability in diagnostic criteria, sample sizes, and measures of oral health further complicates the generalization of findings. Future research should focus on longitudinal and intervention-based studies to clarify the causal mechanisms underlying these associations. A critical comparison of the literature shows that ASD, depression, bipolar disorder, and schizophrenia all present unique challenges to oral health. However, variations in reported outcomes indicate the need for further investigation into how behavioral patterns, medication use, and access to healthcare services influence these conditions. This variability highlights the importance of personalized dental care strategies tailored to the specific needs of each mental health disorder [27,38,41,47]. Such studies could provide valuable insights into how integrating dental care with mental health management can improve both oral and psychological outcomes in psychiatric populations. Recognizing the complex interplay between mental and oral health and developing comprehensive care strategies can help healthcare providers improve the quality of life for individuals with mental illnesses and reduce the burden of oral diseases. Longitudinal studies are essential to better establish causal relationships between mental and oral health and to develop effective interventions that meet the specific needs of these populations.

## 7. Conclusions

In conclusion, the relationship between mental health and oral health is intricate and multifaceted, involving a range of behavioral, cognitive, and physiological factors, as well as social and economic barriers. Addressing these challenges requires a comprehensive approach that integrates mental health management with dental care, ensuring that individuals with mental illnesses receive the support they need to maintain good oral health. By focusing on the unique needs of each patient and developing personalized care strategies, healthcare providers can improve oral health outcomes and overall quality of life for individuals with mental illnesses.

## Figures and Tables

**Table 1:** Comparison of key oral health issues and findings across different mental health disorders.

Mental Disorder	Key Oral Health Issues	Study Findings
ASD [22,26,33,34]	Dental caries, Periodontal disease, Xerostomia	Higher caries prevalence reported due to dietary preferences and hygiene challenges; some studies report comparable outcomes with specialized care
Depression [7,35,40]	Xerostomia, Dental caries, Periodontal disease, Bruxism	Reduced motivation for oral care and xerostomia linked to antidepressant use; significant impact on caries and periodontal disease
Bipolar disorder [41,42,44]	Dental caries, Periodontal disease, Gingival hyperplasia	Alternating manic and depressive phases result in either aggressive hygiene practices or neglect, leading to caries and periodontal issues
Schizophrenia [45,46,47,48]	Dental caries, Periodontal disease, Xerostomia, Tardive dyskinesia	Antipsychotic medication side effects, cognitive impairment, and social barriers contribute to poor oral health outcomes

**Table 2:** Summary of oral health issues, causes, and interventions for different mental disorders.

Mental Disorder	Oral Health Issues	Potential Causes	Suggested Interventions
ASD [22,23,24,25,26,27,28,29,33,34]	Dental caries, Periodontal disease, Open bite, Xerostomia	Sensory sensitivities, preference for soft/sugary foods, medication side effects (antipsychotics causing dry mouth), motor coordination difficulties hindering oral hygiene	- Sensory-friendly dental environments - Desensitization techniques - Caregiver education on daily oral hygiene routines - Use of electric toothbrushes and fluoride varnishes to assist oral care
Depression [2,7,8,10,11,35,36,37,38,39,40]	Dental caries, Periodontal disease, Xerostomia, TMJD	Reduced motivation for self-care, medication side effects (antidepressants causing dry mouth), poor nutrition, altered pain perception, dental anxiety	- Integrated care with mental health management - Saliva substitutes - Fluoride treatments - Dietary counseling - Enhanced oral hygiene support, including oral hygiene motivation strategies
Bipolar disorder [41,42,43,44]	Dental caries, Periodontal disease, Xerostomia, Gingival hyperplasia	Alternating manic (excessive brushing) and depressive (neglecting oral hygiene) behaviors, medication side effects (e.g., lithium causing dry mouth, anticonvulsants causing gum overgrowth)	- Regular monitoring of oral health during mood shifts - Coordination with healthcare providers to adjust medications and manage side effects - Patient education on avoiding overzealous brushing during manic phases
Schizophrenia [1,3,4,12,46,47,48,49,50,51]	Dental caries, Periodontal disease, Xerostomia, Tardive dyskinesia	Cognitive impairments, medication side effects (antipsychotics causing dry mouth and movement disorders), social stigma, financial constraints limiting access to care	- Specialized dental care - Regular check-ups and cleanings to prevent caries and periodontitis - Collaboration between dental and mental health professionals to manage tardive dyskinesia and oral hygiene
Anxiety and depressive disorders [2,8,10,11]	Higher rates of dental caries, periodontal disease, tooth loss	Anxiety-induced bruxism, antidepressant-induced xerostomia, poor oral hygiene from reduced motivation	- Behavioral interventions for managing stress and bruxism - Use of fluoride rinses - Regular dental check-ups - Management of xerostomia with saliva substitutes and routine dental cleaning

**Table 3:** Recommendations and guidelines for managing oral health in individuals with mental disorders.

Group	Recommendations and Guidelines
Healthcare providers	Integrate regular dental check-ups into the mental health care plan, especially for patients taking psychotropic medications that may induce xerostomia [1,7] Educate patients and caregivers on the use of electric toothbrushes and fluoride treatments to maintain oral hygiene [7,30] Collaborate with mental health professionals to provide holistic care and ensure oral health is a part of the overall treatment plan [1,42]
Patients and caregivers	Establish daily oral hygiene routines, including brushing twice a day and using fluoride toothpaste to prevent dental caries [7,11] Use saliva substitutes or prescribed medications to manage dry mouth symptoms caused by psychotropic medications [7] For individuals with conditions like autism spectrum disorder, seek specialized dental care environments to reduce anxiety and improve outcomes [30]

## Data Availability

The data presented in this study are available in this article.
